# Frequency of *kdr* mutations in the voltage-sensitive sodium channel (*V*_*SSC*_) gene in *Aedes aegypti* from Yogyakarta and implications for *Wolbachia*-infected mosquito trials

**DOI:** 10.1186/s13071-020-04304-x

**Published:** 2020-08-24

**Authors:** Juli Rochmijati Wuliandari, Ary A. Hoffmann, Warsito Tantowijoyo, Nancy M. Endersby-Harshman

**Affiliations:** 1 Universitas Muhammadiyah Purwokerto, Jl KH Ahmad Dahlan, Banyumas, 53182 Jawa Tengah Indonesia; 2 Pest and Environmental Adaptation Research Group, School of BioSciences, Bio21 Institute, The University of Melbourne, 30 Flemington Rd, Parkville, Victoria, 3010 Australia; 3 World Mosquito Program Yogyakarta, Centre for Tropical Medicine, Faculty of Medicine, Public Health and Nursing, Universitas Gadjah Mada, Yogyakarta, Indonesia

**Keywords:** Pyrethroid, Insecticide resistance, Dengue

## Abstract

**Background:**

In the inner city of Yogyakarta, Indonesia, insecticide resistance is expected in the main dengue vector, *Aedes aegypti*, because of the intensive local application of pyrethroid insecticides. However, detailed information about the nature of resistance in this species is required to assist the release of *Wolbachia* mosquitoes in a dengue control program, so that we can ensure that insecticide resistance in the strain of *Ae. aegypti* being released matches that of the background population.

**Methods:**

High-resolution melt genotyping was used to screen for *kdr* mutations associated with pyrethroid resistance in the voltage-sensitive sodium channel (*V*_*SSC*_) gene in *Ae. aegypti* of some areas in the inner city of Yogyakarta.

**Results:**

The results show that the V1016G mutation predominated, with individuals homozygous for the 1016G allele at a frequency of 82.1% and the mutant allele G at a frequency of 92%. Two patterns of co-occurrence of mutations were detected in this study, homozygous individuals V1016G/S989P; and heterozygous individuals V1016G/F1534C/S989P. We found the simultaneous occurrence of *kdr* mutations V1016G and F1534C at all collection sites, but not within individual mosquitoes. Homozygous mutants at locus 1016 were homozygous wild-type at locus 1534 and *vice versa*, and heterozygous V1016G were also heterozygous for F1534C. The most common tri-locus genotype co-occurrences were homozygous mutant 1016GG and homozygous wild-type FF1534, combined with homozygous mutant 989PP (GG/FF/PP) at a frequency of 38.28%.

**Conclusions:**

Given the relatively small differences in frequency of resistance alleles across the city area, locality variations in resistance should have minor implications for the success of *Wolbachia* mosquito trials being undertaken in the Yogyakarta area.

## Background

Dengue is the most important mosquito-borne viral disease in the world [1]. Dengue is also a significant public health problem in Indonesia since the first reported dengue outbreak in 1968. All four dengue virus (DENV, *Flavivirus*) serotypes are detected, suggesting hyperendemicity in all of 34 provinces in Indonesia [2, 3]. Dengue virus is transmitted by *Aedes aegypti*, a mosquito that has also been linked with the transmission of other major arboviral diseases including chikungunya (CHIKV) and Zika (ZIKV). The virologically confirmed chikungunya was initially reported during an outbreak in Jambi in 1982 [4]. After nearly 20 years of absence, twenty-four distinct outbreaks of probable chikungunya re-emerged in South Sumatera, Aceh and West Java in the early 2000s [4]. To date, there has been no recorded fatality related to CHIKV infection in Indonesia [5]. Zika virus (ZIKV, family *Flaviviridae*) has become one of the global public health threats because of its association with Guillain-Barré syndrome and microcephaly. Until recently, most evidence for Zika virus infection reported in Asia, including in Indonesia, has been serological [6].

Indonesia has made dengue a notifiable disease. On average, 136,670 DENV infections and 1112 deaths were reported annually from 2013 to 2016, an incidence of about 54 dengue fever or dengue hemorrhagic fever cases per 100,000 population and a case fatality rate of approximately 1% [7]. Dengue cases in Indonesia are expected to be under-reported due to poor disease surveillance and a low level of reporting [8]. The surveillance database likely covers dengue probable cases with supportive dengue serology or with epidemiologic linkage, and dengue confirmed cases with confirmatory laboratory criteria [8, 9]. Dengue infections deprived of diagnostic tests may not end up in the surveillance database [8]. Despite the level of uncertainty on total case number, dengue visibly results in a considerable cost to the health sector, and a heavy economic and social impact is likely.

Indonesia has made progress in many areas of dengue prevention and control. In August 2016, Indonesia became the sixth country to approve the first licensed dengue vaccine, Dengvaxia® [10, 11]. To date, the vaccine so far has been approved in 20 countries and launched in 11, including Indonesia [11]. However, in December 2017, the National Agency of Drug and Food (Badan POM) suspended the use of dengue vaccine in Indonesia over safety concerns [12] and later in February 2018, the BPOM revised recommendation that the use of vaccine be limited for people between 9–16 years of age who had a dengue infection prior to vaccination and that the vaccine should not be administered to people who have never had a dengue infection before [13]. There is no rapid, reliable test for previous dengue infection, so the Dengvaxia® vaccine cannot be widely used [14]. Nevertheless, effective vector control methods are still essential, targeting *Ae. aegypti* in its immature and adult stages. It has been argued that even if the current vaccine is highly targeted and low-cost, sustained mosquito control will remain cost-effective [15].

The mainstay of the current vector control programme in Indonesia is environmental management, which in recent years, has emphasized community participation to reduce container breeding sites [2, 16]. The country is renowned for its 3M plus campaigns, aimed at covering and cleaning water containers and burying discarded water containers, complemented with biological approaches using natural predators or pathogens as alternatives for vector control [2, 16]. The efficacy of community-based approaches is measured by a larvae-free index (percentage of houses free from *Ae. aegypti* larvae and pupae infestation); unfortunately, data from the last five years showed a larvae-free index ranging between 24–80%, less than the target of 95% [7].

Chemical insecticides still play a central role in dengue vector control in Indonesia. Vector control during outbreaks depends primarily on chemical insecticides to effect a rapid reduction in the number of infected mosquitoes and to break the dengue transmission cycle [2, 16–18]. Since the 1970s, the organophosphates malathion and temephos have been widely used to control dengue, and starting in the 1980s, dengue vector control has been highly reliant on pyrethroids. Pyrethroids are also widely used in public health for prevention and control of other mosquito-borne diseases such as malaria and filariasis, and as agricultural insecticides [19]. Pyrethroids are also approved for household protection against dengue [20, 21]. The abundant and prolonged use of pyrethroids has led to the development of resistance in *Ae. aegypti* populations in many countries including Indonesia. Resistance to pyrethroids, based on bioassay data, has been reported in some areas of Indonesia [22–26].

Two main mechanisms for pyrethroid resistance have been identified in *Ae. aegypti*, metabolic resistance and target site resistance. Metabolic resistance occurs when increased levels or modified activities of one or more detoxifying enzymes result in a more rapid detoxification of the insecticide, preventing the insecticide from reaching its target in the nervous system. Metabolic resistance involves three groups of enzymes, i.e. esterases, multi-function oxidases P450 and glutathione s-transferases (GST) [27–29]. Limited studies about metabolic resistance based on biochemical assays are available for Indonesian dengue vectors [26, 30–32].

Pyrethroids act on the insect nervous system, targeting the voltage-sensitive sodium channel (*V*_*SSC*_). They modify the gating kinetics of the channel by slowing both the activation and the inactivation, stimulating the nerve cells to produce repetitive discharge that lead to paralysis and death of insects, an effect known as knockdown. Target site resistance is caused by point mutations in the *V*_*SSC*_ gene, resulting in amino acid substitutions that affect pyrethroid binding sites, being known as *kdr* (knockdown resistance) mutations [33, 34]. A total of 12 point mutations in the *V*_*SSC*_ of *Ae. aegypti* have been identified to be associated with *kdr* resistance to pyrethroids. Only five of these mutations have been functionally confirmed to reduce the sensitivity of mosquito sodium channels to pyrethroids, i.e. S989P, I1011M, V1016G/I, F1534C, and recently V410L [35]. The mutations L982W, G923V, I1011M and V1016G were the first reported sodium channel mutations in permethrin/DDT-resistant populations of *Ae. aegypti* from various countries [36], located in the domain IIS5 (for L982W and G923 mutations) and IIS6 (for I1011M and V1016G mutations) of the *V*_*SSC*_ [36]. Further studies have reported novel mutations, including I1011V and V1016I in Latin American populations [37]. In Asian countries, the F1534C mutation (in domain IIIS6) was detected in *Ae. aegypti* mosquitoes from Thailand [38] and Vietnam [39]. The S989P mutation, located in linker between domains IIS5–S6, was first reported in *Ae. aegypti* populations in Thailand [40], the D1763Y mutation (in linker IVS5-S6) was reported in a Taiwan population [41], and mutation T1520I (in domain IIS6) was detected in an Indian population [42]. In 2017, the mutation V410L was first identified in Brazilian strains of *Ae. aegypti* [43], and in 2018, a novel mutation V419L was found in populations from Colombia [44] (both mutations are located in domain IS6).

Three of these mutations, V1016G, F1534C and S989P, are widely distributed and detected in pyrethroid-resistant populations in Southeast Asian countries including Thailand, Indonesia, Malaysia, Singapore, Vietnam, Cambodia and Laos [45]. Co-occurrence of *kdr* mutations has been a common phenomenon observed and, for some combinations, has been shown to confer a higher level of resistance than singly occurring mutations [46]. At least three patterns of mutational associations have been identified in *Ae. aegypti* from Southeast Asia: V1016G/F1534C, V1016G/S989P and V1016G/F1534C/S989P. Co-occurrence of V1016G and F1534C was reported in populations from Thailand [47], Myanmar [46], Malaysia [48] and Indonesia [49–52] and co-occurrence of V1016G and S989P point mutations was detected in Thailand [47], Myanmar [46], Indonesia, [49, 50, 52] and Papua New Guinea [53]. In addition, co-occurrence of triple mutations V1016G/F1534C/S989P in heterozygous form has been identified commonly in *Ae. aegypti* from Thailand [54], Myanmar [46] and Indonesia [49, 50, 52]. However, co-occurrence of triple homozygous point mutations (homozygous mutation for each of V1016G, F1534C and S989P) is very rare having only been reported from Myanmar at a frequency of 0.98% [46] and in Indonesia in one individual (at a frequency of 0.34%) [50]

Despite all of the strategies implemented in Indonesia, the existing methods of controlling dengue have limited success. The development of a novel strategy of vector control that can be incorporated into the existing vector control strategy is essential. One of the innovative approaches to preventing transmission of dengue virus involves introduction of strains of the bacterium *Wolbachia* into *Ae. aegypti*, which has both life-shortening effects on the mosquito and direct transmission-blocking effects on dengue virus. This new strategy, together with more traditional approaches for vector control including insecticide application, may provide promising results to reduce dengue transmission. With the support of communities and approval from regulators, Indonesia’s first field trial of *Wolbachia*-infected mosquitoes began in Yogyakarta in 2014 [55]. The first field trial in two areas in the outer city of Yogyakarta has yielded results that point to local invasion [56] and release of *Wolbachia-*infected mosquitoes on a broader scale in the inner city areas of Yogyakarta has since been initiated in August 2016 [57].

**Fig. 1:**
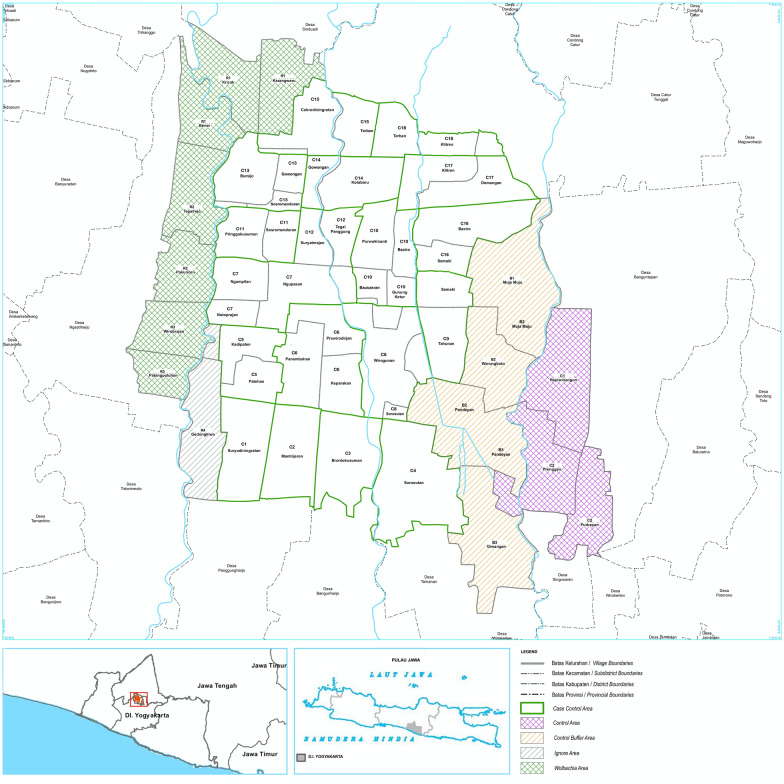
Sample collection sites for *Aedes aegypti* from inner city of Yogyakarta, Indonesia. The map was sourced from ‘Map of case control area borders, city of Yogyakarta, Eliminate Dengue Project Yogyakarta’

To assist the spread of *Wolbachia,* insecticide resistance in the strain of *Ae. aegypti* being released, needs to match that of the background population to ensure that released individuals persist and reproduce, allowing a *Wolbachia* invasion to take place [58]. In the inner city of Yogyakarta, resistance is expected because of the local heavy application of chemical insecticides. It is possible that insecticide usage is higher in these areas than in the outer rim of Yogyakarta, as inner city areas have more dengue cases [59] and are more densely populated [60]. In a previous study, we screened samples from Yogyakarta outer areas for *kdr* mutations and obtained a high frequency of *kdr* alleles in samples collected from the outer area of Yogyakarta [49]. The most common *kdr* co-occurrence was V1016G homozygous mutant/F1534 wild type, combined either with S989P heterozygous, or S989P wild type, or S989P homozygous mutant, respectively. This co-occurrence was more common in the surviving than in the dead mosquitoes [49] and a strong association between the V1016G mutation with pyrethroid resistance (type I and type II) was confirmed. In contrast, F1534C mutant homozygotes were rare and there was only a weak association between heterozygote individuals for the F1534C mutation and resistance to a type I pyrethroid. The S989P mutation, in addition to the V101G mutation, was found to have an additive effect in resistance to Type II pyrethroids [49]. In this study, we have screened Yogyakarta inner city areas for *kdr* mutations, based on a total of 1314 individuals collected from 27 localities (Fig. 1, Table 1) and aim to compare frequency and occurrence of *kdr* mutations between the inner and outer city.

**Table 1:** Collection sites, localities and number of *Aedes aegypti* collected from inner city areas of Yogyakarta

Site	Locality	No. of samples	Geolocation coordinates(longitude, latitude)	
C1	Suryodinigratan	112	110.35971	− 7.81972
C2	Mantrijeron	25	110.36539	− 7.82453
C3	Brontokusuman	25	110.36996	− 7.82397
C4	Sorosutan	124	110.37840	− 7.82886
C5	Kadipaten	34	110.35872	− 7.80587
	Patehan		110.36015	− 7.81104
C6	Panembahan	25	110.36486	− 7.81063
	Keparakan		110.37327	− 7.81464
	Prawirodirjan		110.37287	− 7.80581
C7	Notoparajan	140	110.35560	− 7.80621
	Ngampilan		110.35577	− 7.79728
	Ngupasan		110.36199	− 7.80199
C8	Wirogunan	25	110.37810	− 7.81130
	Sorosutan		110.37872	− 7.81484
C9	Tahunan	19	110.38273	− 7.81056
C10	Bausasran	25	110.37448	− 7.79253
	Gunung Ketur		110.37883	− 7.80142
	Baciro		110.37875	− 7.79241
	Purwokinanti		110.37382	− 7.79717
C11	Pringgokkusuman	21	110.36969	− 7.79702
	Sosromenduran		110.36458	− 7.79287
C12	Suryatmajan	124	110.36969	− 7.79702
	Tegal Panggung		110.36983	− 7.79350
C13	Bumijo	25	110.35676	− 7.78798
	Sosromenduran		110.36460	− 7.78835
	Gowongan		110.36467	− 7.78353
C14	Gowongan	25	110.36921	− 7.78485
	Kotabaru		110.36935	− 7.78920
C15	Cokrodiningaratan	88	110.36914	− 7.77882
	Terban		110.37384	− 7.77877
C16	Semaki	68	110.38617	− 7.79694
	Baciro		110.38211	− 7.79685
C17	Klitren	25	110.38212	− 7.78394
	Demangan		110.38219	− 7.78927
C18	Terban	25	110.37876	− 7.77835
	Klitren		110.38331	− 7.78029
R1	Bener	31	110.352325	− 7.77969
	Kricak		110.360298	− 7.77916
	Karangwaru		110.364609	− 7.77532
R2	Tegalrejo	38	110.35648	− 7.78339
	Pakuncen		110.35070	− 7.79298
R3	Wirobrajan	49	110.35088	− 7.80139
	Patangpuluhan		110.34646	− 7.80694
R4	Gedongkiwo	32	110.35344	− 7.82552
B1	Muja Muju	27	110.39291	− 7.79768
B2	Muja Muju	87	110.39259	− 7.80637
	Warung Boto		110.38804	− 7.81086
	Pandeyan		110.38647	− 7.81439
B3	Pandeyan	24	110.38669	− 7.82032
	Giwangan		110.39137	− 7.83290
Co1	Rejojwinangun	39	110.40043	− 7.81836
Co2	Prenggan, Purbayan	36	110.39775	− 7.82482

*Notes*: Localities are mapped in Fig. 1. The clusters refer to areas that are being used in a controlled intervention design to investigate the impact of *Wolbachia* on dengue incidence by the World Mosquito Programme (Indonesia). Areas marked by an R are not included as part of this design

## Methods

### Mosquito samples

*Aedes aegypti* samples (male and female adults) were collected in June-July 2015 using BioGent-Sentinel (BG-S) traps (Biogents AG, Regensburg, Germany), each locality was covered by several BG-traps. Samples were collected every week and identified to species level and *A. aegypti* were preserved in absolute ethanol.

### High-resolution melt (HRM) genotyping of *kdr* mutations in *Ae. aegypti* samples from Yogyakarta

A total of 1293 samples from 27 collection sites were screened for *kdr* mutations at 1016, 1534 and 989 following the HRM assay protocol of our previous study [49]. The PCR primer pairs, the region amplified in *V*_*SSC*_ and the product size are shown in Table 2.

**Table 2:** Primers to amplify *V*_*SSC*_ mutations of *Aedes aegypti* from the city of Yogyakarta, Indonesia

**Mutation**	**Region amplified**	**Sequence (5’-3’)**	**Product size (bp)**
V1016G	IIS6 first codon of exon 21	GACAAATTGTTTCCCACCCGCACAG	52
		AAGCAAGGCTAAGAAAAGGTTAAG	
F1534C	IIIS6 24th codon of exon 31	TACCTCTACTTTGTGTTCTTCATCATC	52
		GATTCAGCGTGAAGAACGACCCG	
S989P	IIS5 S6 first codon of P region	CGGGTATTATGCGGCGAGTGGATC	52
		CCCACAAGCATACAAT CCCACATGG	

We used the High Pure PCR Template Preparation Kit (Roche, Mannheim, Germany) to extract mosquito DNA in a final volume of 200 μl of elution buffer and prepared a 10-fold dilution of the template DNA. PCR reactions (10 μl) contained 2 μl of 1 in 10 diluted template DNA, 0.4 μl each of the primers at 10 μM, 1 μl of the ThermoPol reaction buffer (NEB Inc., Ipswich, MA, USA), 0.064 μl of dNTPs at 25 mM (Bioline, Alexandria, NSW, Australia), 0.4 μl of MgCl_2_ (50 mM) (Bioline, Alexandria, NSW, Australia), 0.25 μl of the LightCycler® 480 High Resolution Melting Master (Roche, Mannheim, Germany), 0.01 μl of IMMOLASE^TM^ DNA polymerase (10 u/μl) (Bioline, Alexandria, NSW, Australia) and 5.476 μl of ddH_2_O (Honeywell, Burdick and Jackson; Muskegon, MI, USA).

Amplification was performed on a Roche LightCycler® 480 system (384-well format) using the following temperature cycling conditions: 95 °C for 10 min, 20 cycles of 95 °C for 5 s, 65 °C (reduce 0.5 °C each cycle) for 15 s, 72 °C for 15 s, followed by an additional 20 cycles of 95 °C for 5 s, 55 °C for 15 s and 72°C for 15 s. Fluorescence information was captured at the end of each 72 °C step. Following amplification, PCR products were subjected to HRM analysis. The HRM step involved heating the PCR products to 95 °C for 1 min, cooling to 40 °C for 20 s and then increasing the temperature to 65 °C and then melting at 0.05 °C/s with continuous acquisition of fluorescence as the temperature increased from 65 to 95 °C. Melt curves were generated in the Gene Scanning module of the Roche LightCycler® 480 software package. Melting data were normalized, temperature shifted, and displayed as derivative curves compared to the known genotyped samples. We used the same PCR protocol and thermocycling conditions, and HRM step for all *kdr* mutations except the parameter settings for melt curve normalization.

### Statistical analysis

Statistical comparisons of locations for genotypic frequencies for the V1016G, F1534C and S989P loci were undertaken using a Chi-square contingency test (IBM SPSS version 22) with degrees of freedom dictated by the number of localities being compared. We also tested for spatial signature in the data using the average GPS points for the BG traps in a locality and then computing Moran’s I using the software Spatial Analysis in Macroecology [61].

Hardy-Weinberg equilibrium for observed genotyped frequencies for each *kdr* mutation was calculated using GenAlEx 6.5 (http://biology.anu.edu.au/GenAlEx/) [62, 63]. Linkage disequilibrium between the two-*kdr* mutations, V1016G and F1534C, was also tested. Data were pooled across adjacent sites to increase sample size for detecting linkage disequilibrium.

We identified three patterns of mutational associations: (i) nearly all homozygous mutant individuals 1016GG occurred in conjunction with homozygous wild-type 1534FF; (ii) homozygous mutant individuals 1534CC occurred with the wild-type 1016VV; and (iii) heterozygous individuals for V1016G were commonly heterozygous for F1534C. We used Pearson’s correlation (MS Excel v.16.23) to measure the strength of relationship of these mutational associations.

## Results

### HRM genotyping of *kdr* mutations in *Ae. aegypti* samples from Yogyakarta

A total of 1314 of *Ae. aegypti* were collected from 27 different sites in the inner city of Yogyakarta (Fig. 1, Table 1). Of these, we genotyped 1293 for *kdr* mutations at V1016G, F1534C and S989P sites using the high-resolution melt (HRM*)* protocol [49] and compared these according to their geographical location (Fig. 1). V1016G was the most widespread *kdr* point mutation and was detected in all collection sites: 82.06% of the individuals had the homozygous mutant genotype 1016GG, 16.86% were heterozygous 1016VG and the remaining 1.08% were homozygous wild type 1016VV (Table 3). The allelic frequencies of V1016 and 1016G were 0.105 and 0.895, respectively. All collection sites displayed a high frequency of the mutant allele 1016G, ranging between 0.760–1.000. The observed frequency of the mutant allele 1016G was statistically different between collection sites as indicated by a Chi-square contingency test ($\chi_{(26)}^{2}$ = 44.145, *P* = 0.015). For the 1016 allele frequencies, Moran’s I was not significant for any distance class, indicating locality differences unrelated to distance.

**Table 3:** V1016G, F1534C and S989P genotypes of *Aedes aegypti* samples from Yogyakarta and Chi-square tests for Hardy-Weinberg equilibrium

Collection code	Collection site	*n*	*kdr* mutations	SS	RS	RR	Frequency of R allele (95% CI)	*χ*^2^	*P*
C1	Suryodiningratan	110	V1016G	1	21	88	0.896 (0.848–0.929)	0.042	0.837
			F1534C	89	20	1	0.100 (0.067–0.147)	0.011	0.916
			S989P	23	47	40	0.577 (0.511–0.641)	1.706	0.191
C2	Mantrijeron	25	V1016G	0	4	21	0.920 (0.812–0.969)	0.189	0.664
			F1534C	21	4	0	0.080 (0.032–0.188)	0.189	0.664
			S989P	3	17	5	0.540 (0.414–0.670)	3.400	0.065
C3	Brontokusuman	25	V1016G	0	5	20	0.900 (0.786–0.957)	0.309	0.664
			F1534C	20	5	0	0.100 (0.044–0.214)	0.309	0.664
			S989P	6	13	6	0.500 (0.333–0.634)	0.040	0.065
C4	Sorosutan	122	V1016G	0	9	113	0.963 (0.931–0.981)	0.198	0.656
			F1534C	114	8	0	0.033 (0.017–0.063)	0.198	0.656
			S989P	15	57	50	0.643 (0.582–0.701)	2.258	0.133
C5	Kadipaten, Patehan, Panembahan	30	V1016G	0	6	24	0.900 (0.781–0.953)	0.370	0.543
			F1534C	24	6	0	0.100 (0.047–0.216)	0.370	0.543
			S989P	1	16	13	0.700 (0.575–0.801)	2.184	0.139
C6	Panembahan, Keparakan, Prawirodirjan	24	V1016G	0	4	20	0.917 (0.805–0.967)	0.023	0.878
			F1534C	20	4	0	0.083 (0.033–0.196)	0.004	0.709
			S989P	2	15	7	0.604 (0.463–0.730)	0.002	0.272
C7	Notoprajan, Ngupasan, Ngampilan	136	V1016G	1	23	112	0.908 (0.868–0.937)	0.140	0.709
			F1534C	113	22	1	0.088 (0.060–0.128)	0.140	0.709
			S989P	15	60	61	0.669 (0.611–0.722)	0.851	0.356
C8	Wirogunan, Sorosutan	25	V1016G	2	8	15	0.760 (0.626–0.857)	1.205	0.272
			F1534C	20	3	2	0.140 (0.070–0.262)	1.205	0.272
			S989P	6	16	3	0.440 (0.312–0.577)	1.340	0.247
C9	Tahuna	19	V1016G	0	3	16	0.921 (0.792–0.921)	0.416	0.519
			F1534C	16	3	0	0.079 (0.027–0.208)	0.416	0.519
			S989P	2	11	6	0.605 (0.447–0.744)	0.012	0.914
C10	Bausasran, Gunung Ketur, Baciro, Purwokinanti	25	V1016G	1	5	19	0.860 (0.738–0.931)	na	
			F1534C	20	4	1	0.120 (0.056–0.238)	na	
			S989P	11	10	4	0.360 (0.241–0.499)	1.995	0.158
C11	Pringgokussuman, Sosromenduran	21	V1016G	0	0	21	1.000 (0.916–1.000)	1.574	0.210
			F1534C	21	0	0	0.000 (0.000–0.084)	1.574	0.210
			S989P	5	7	9	0.595 (0.445–0.730)	0.884	0.347
C12	Suryatmajan, Tegal Panggung	123	V1016G	0	25	98	0.898 (0.854–0.930)	0.107	0.744
			F1534C	98	25	0	0.102 (0.070–0.146)	0.107	0.744
			S989P	13	61	49	0.646 (0.585–0.703)	0.160	0.689
C13	Bumijo, Sosromenduran, Gowongan	24	V1016G	0	3	21	0.938 (0.832–0.979)	1.361	0.243
			F1534C	21	3	0	0.063 (0.022–0.168)	1.361	0.243
			S989P	7	11	6	0.479 (0.345–0.617)	1.434	0.231
C14	Gowongan, Kotabaru	24	V1016G	1	4	19	0.875 (0.753–0.941)	0.718	0.397
			F1534C	19	4	1	0.125 (0.059–0.247)	1.469	0.225
			S989P	2	14	8	0.625 (0.484–0.748)	0.435	0.509
C15	Cokrodiningratan, Terban	88	V1016G	1	12	75	0.921 (0.871–0.952)	0.377	0.539
			F1534C	75	12	1	0.080 (0.048–0.129)	6.292	0.012
			S989P	10	40	38	0.659 (0.586–0.725)	2.231	0.135
C16	Semaki-Baciro	68	V1016G	1	12	55	0.897 (0.835–0.938)	0.135	0.714
			F1534C	55	12	1	0.103 (0.062–0.165)	0.135	0.714
			S989P	8	37	23	0.610 (0.526–0.692)	1.408	0.235
C17	Klitren, Demangan	24	V1016G	0	5	19	0.896 (0.778–0.955)	0.324	0.569
			F1534C	19	5	0	0.104 (0.045–0.222)	0.324	0.569
			S989P	6	10	8	0.542 (0.403–0.674)	0.621	0.431
C18	Terban-Klitren	25	V1016G	0	9	16	0.820 (0.692–0.921)	0.179	0.672
			F1534C	16	9	0	0.180 (0.098–0.308)	0.140	0.708
			S989P	3	15	7	0.580 (0.442–0.706)	0.041	0.840
R1	Karangwaru, Kricak-Bener	30	V1016G	0	5	25	0.920 (0.819–0.964)	0.248	0.619
			F1534C	26	4	0	0.067 (0.026–0.159)	0.153	0.696
			S989P	4	13	13	0.605 (0.447–0.744)	0.068	0.794
R2	Tegalrejo, Pakuncen	38	V1016G	2	7	29	0.855(0.759–0.917)	2.489	0.115
			F1534C	29	7	2	0.145 (0.083–0.241)	2.489	0.115
			S989P	5	15	18	0.671 (0.660–0.766)	0.426	0.514
R3	Wirobrajan, Patangpuluhan	46	V1016G	0	9	37	0.902 (0.825–0.948)	0.541	0.462
			F1534C	37	9	0	0.098(0.052–0.176)	0.541	0.462
			S989P	6	19	21	0.663 (0.562–0.751)	0.263	0.608
R4	Gedongkiwo	32	V1016G	0	4	28	0.938 (0.850–0.975)	0.142	0.706
			F1534C	28	4	0	0.063(0.025–0.150)	0.142	0.706
			S989P	7	9	16	0.641(0.518–0.747)	4.847	0.028
B1	Muja Muju	27	V1016G	0	7	20	0.870 (0.756–0.936)	0.600	0.439
			F1534C	20	7	0	0.130 (0.064–0.244)	0.600	0.439
			S989P	4	12	11	0.630 (0.496–0.746)	0.600	0.807
B2	Muja Muju, Warungboto, Pandeyan	85	V1016G	0	17	68	0.900 (0.846–0.937)	1.049	0.306
			F1534C	68	17	0	0.100 (0.063–0.154)	1.049	0.306
			S989P	12	41	32	0.618 (0.543–0.687)	0.038	0.845
B3	Pandeyan, Giwangan	24	V1016G	0	1	23	0.979 (0.891–0.996)	0.011	0.917
			F1534C	23	1	0	0.021 (0.004–0.109)	0.011	0.917
			S989P	0	15	9	0.688 (0.547–0.801)	4.959	0.026
Co1	Rejowinangun	39	V1016G	4	4	31	0.846 (0.750–0.910)	14.325	< 0.0001
			F1534C	33	3	3	0.115 (0.062–0.205)	15.146	< 0.0001
			S989P	7	13	19	0.652 (0.543–0.756)	2.710	0.100
Co2	Prenggan, Purbayan	34	V1016G	0	6	28	0.912 (0.821–0.959)	0.318	0.573
			F1534C	28	6	0	0.088 (0.041–0.179)	0.318	0.573
			S989P	7	14	13	0.588 (0.470–0.697)	0.765	0.382
Total		1293	V1016G	14	218	1061	0.915 (0.893–0.916)	0.552	0.458
			F1534C	1073	207	13	0.080 (0.026–0.102)	0.721	0.396
			S989P	190	608	495	0.612 (0.599–0.637)	0.022	0.882

*Abbreviation*: na, not avalable

The second most common *kdr* mutation detected in our samples was S989P. The frequencies of genotypes detected overall were as follows: homozygous wild-type 989SS, 14.54%; heterozygous 989SP, 47.18%; and homozygous mutant 989PP, 38.28%. Allelic frequencies overall were 0.599 for S989 and 0.401 for 989P. The mutant allelic frequency 989P ranged between 0.360–0.700 across localities and overall there was a statistically significant difference between localities (Chi-square contingency test, $\chi_{(26)}^{2}$= 43.473, *P* = 0.017). For the 989 allele frequencies, Moran’s I did not show a significant departure from randomness at the smallest distance class (0.85 km), but there was a positive association at 1.7 km and negative association at the next class up (2.2 km), suggesting a complex pattern not related directly to distance.

For the 1534 site, the homozygous wild-type genotype 1534FF was the most common at 83.06%, while the homozygous mutant genotype 1534CC was rare at 1.00% and heterozygous genotype 1534F/C was at 15.94%. A contingency test indicates no significant difference between the localities in allele frequencies ($\chi_{(26)}^{2}$= 32.151, *P* = 0.188). Moran’s I was not significant for any distance classes for the 1534 alleles.

All genotypes at the three loci in all field collections were in Hardy-Weinberg equilibrium except for Co1 (Rejowinangun) (Table 3). When combined across sites, genotype frequencies were also in Hardy-Weinberg equilibrium (Table 4).

**Table 4:** Linkage disequilibrium coefficients (R_ij_) and *χ*^2^ (*df* = 1) for pairwise tests of V1016G and F1534C in *Aedes aegypti* in pooled sites (listed in Table 1)

**Pooled sites**	**R**_**ij**_	***χ***^**2**^	***P***
C1+C2	0.979	129.331	0.001
C3+C4	0.960	135.481	0.001
C5+C6+C7	0.967	177.719	0.001
C8+C9+C10	0.863	51.336	0.001
C11+C12	1.000	144.000	0.001
C13+C14+C15	1.000	136.000	0.001
C16+C17+C18	1.000	117.000	0.001
R1+R2	0.969	63.886	0.001
R3+R4	1.000	78.000	0.001
B1+B2+B3	1.000	136.000	0.001
Co1+Co2	0.880	56.561	0.001

### Mutation combinations

**Fig. 2:**
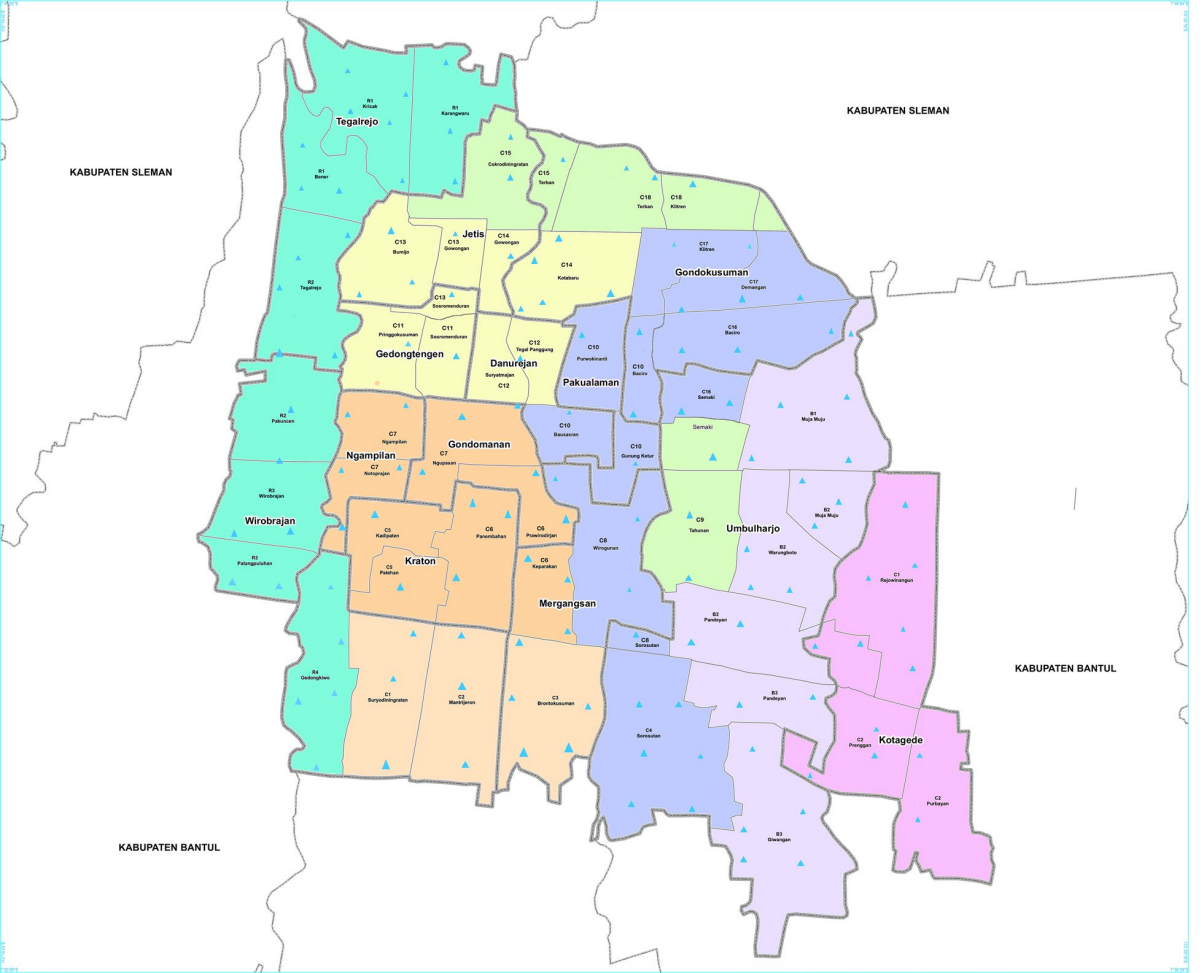
BG trap deployment (mark as triangles) at sample collection sites for *Aedes aegypti* from inner city of Yogyakarta, Indonesia. The map was sourced from ‘Map of case control area borders, city of Yogyakarta, Eliminate Dengue Project Yogyakarta’

We observed that there were nine out of 27 possible genotype combinations (three genotypes at three loci) in our 1293 individual mosquitoes (Fig. 2). Figure 2 shows the frequency of each of the nine tri-locus combinations. The most common tri-locus genotypes were the homozygous mutant 1016GG and homozygous wild-type FF1534, combined with either the homozygous mutant 989PP (GG/FF/PP) (38.28%), the heterozygous S989P (GG/FF/SP) (35.43%), or the homozygous wild-type SS989 (GG/FF/SS) (8.35%). These genotypes totalled 495, 458 and 108 mosquitoes, respectively. Triple mutations were found only in the heterozygous genotype state (VG/FC/SP) at a frequency of 11.14% (144 individuals).

The V1016G homozygote mutant genotype was found alone (GG/FF/SS) in 108 (8.35%) individuals, and there were 8 (1.00%) individuals exhibiting the homozygous mutant genotype for F1534C (VVCCSS). S989P was only found in conjunction either with V1016G (as a homozygote or heterozygote) or F1534C (only as a heterozygote). We only obtained one wild- type individual for the three mutations (VV/FF/SS), likewise there was no individual that expressed triple mutations (homozygous mutants for each site V1016G, F1534C and S989P) among the 1293 mosquito individuals analysed.

**Fig. 3:**
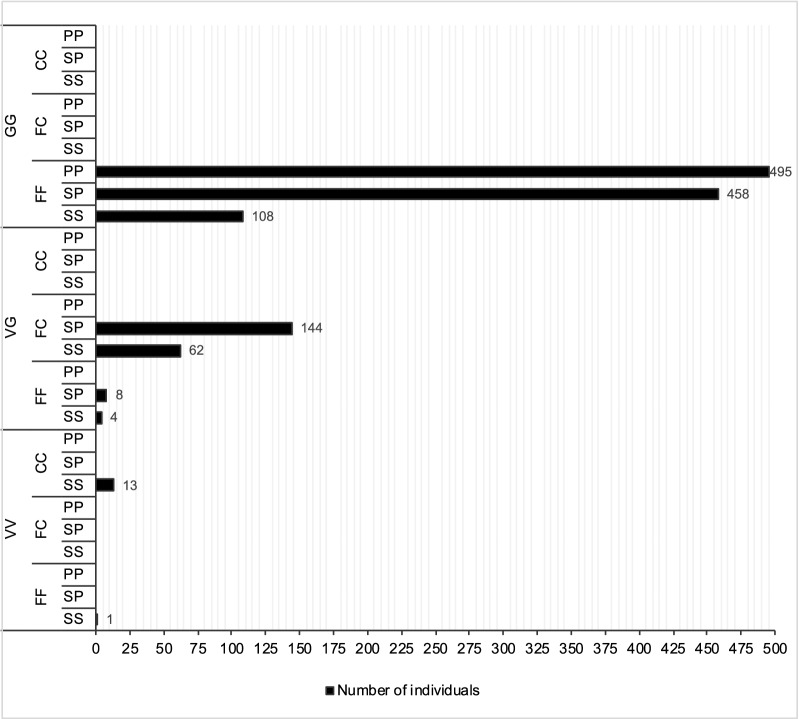
The combinations of *kdr* mutations V1016G (T/G) F1534C (T/G) and S989P (T/C) in *Aedes aegypti* samples collected from the inner city of Yogyakarta

**Fig. 4:**
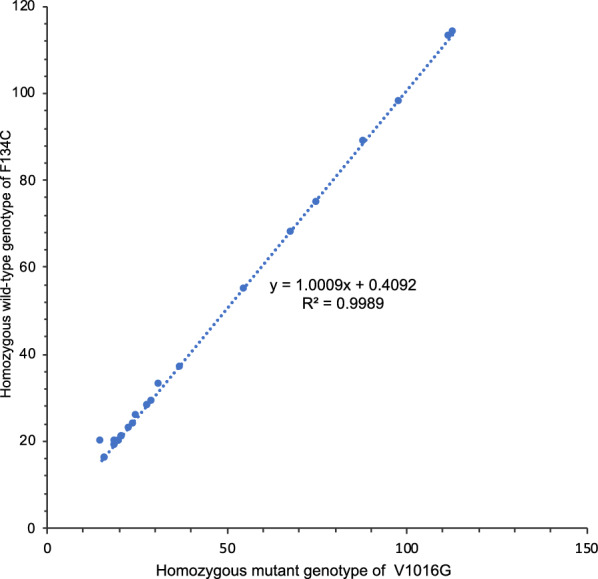
Correlation between the number of *Aedes aegypti* individuals with homozygous mutant genotype V1016G and those with homozygous wild-type genotype F1534C across sample areas

Interestingly, we found that nearly all homozygous mutant 1016GG individuals occurred in conjunction with homozygous wild-type 1534FF, and homozygous mutant individuals 1534CC occurred with the wild-type 1016VV. Heterozygous individuals for V1016G were commonly heterozygous for F1534C, although there was a small number of combined homozygous/heterozygous genotypes (Table 3, Fig. 2). Pearson’s correlation was used to assess these relationship patterns and there was a significant positive correlation between co-occurrence of 1016G homozygous mutants and F1534 wild-type at all collection sites (*r* = 0.999, *n* = 27, *P* < 0.0005), with 1016G homozygous mutants explaining 99.8% of the variation in V1016G mutations (Fig. 3). A strong positive correlation was also found between the co-occurrence of individuals with heterozygous V1016G and heterozygous F1534C (*r* = 0.988, *n* = 27, *P* < 0.0005) (Fig. 4). The most common genotype for V1016C and F1534C was the homozygous mutant genotype 1016GG combined with the homozygous wild-type 1534FF with a frequency of 82.1%, followed by the double heterozygous 1016VG with 1534FC which had a frequency of 9.74% and finally the homozygous wild-type 1016VV combined with the homozygous mutant 1534CC which had a frequency of 1.01%. However, there were 12 individuals with heterozygous 1016VG that were combined with homozygous wild-type 1534FF. Table 4 shows the linkage disequilibrium coefficients (R_ij_), chi-square and associated probabilities obtained between pairwise loci. To calculate linkage disequilibrium, samples from adjacent collection sites were pooled. In all pooled collection sites, there was very strong linkage disequilibrium between V1016G and F1534C, with coefficients (R_ij_) ranged from 0.863 to 1.

We also compared V1016G, F1534C and S989P from those sites where data were available from our previous study in 2012 [49] with this study in 2015. Most allele frequencies were similar between the two sampling periods and did not differ significantly by contingency tests, but there was a significant difference at Cokrodiningratan for S989 ($\chi_{(1)}^{2}$ = 9.88, *P* = 0.002) where frequency of the mutant 989P increased (Table 5).

**Table 5:** Comparison of V1016G/F1534C/S989P in *Aedes aegypti* collected from three sites in 2012/2015 in Yogyakarta-Indonesia

**Site**	**Year**	**Locus**	**SS**	**SR**	**RR**	**Frequency of R allele**
Cokrodiningratan	2012	V1016	0	5	35	0.938
	2015	V1016	1	12	75	0.921
	2012	F1534	37	3	0	0.038
	2015	F1534	75	12	1	0.080
	2012	S989	12	20	8	0.450
	2015	S989	10	40	38	0.659
Purwokinanti	2012	V1016	1	10	27	0.842
	2015	V1016	1	5	19	0.860
	2012	F1534	27	11	0	0.145
	2015	F1534	20	4	1	0.120
	2012	S989	18	18	4	0.488
	2015	S989	11	10	4	0.360
Mantrijeron	2012	V1016	1	5	32	0.908
	2015	V1016	0	4	21	0.920
	2012	F1534	32	6	0	0.079
	2015	F1534	21	4	0	0.080
	2012	S989	14	17	9	0.438
	2015	S989	3	17	5	0.540

## Discussion

The diagnostic identification of the *kdr* mutations in *Ae. aegypti* natural populations is nowadays an important tool to predict resistance to pyrethroids in the field. In this study, we genotyped *kdr* mutations (F1534C, V1016G and S989P) that have been confirmed as being associated with pyrethroid resistance in the *V*_*SSC*_ of *Ae. aegypti* [49, 50, 64, 65]. The homozygous V1016G mutation predominated, with individuals homozygous for the 1016G allele being at a frequency of 82.1% and the mutant allele (G) being at a frequency of 92%. The high frequency of the mutant allele is similar to previous findings for *Ae. aegypti* samples from the outer region of Yogyakarta [49], and some other provinces in Indonesia, including Denpasar (70% in a resistant group and 50% in susceptible groups [52], Jakarta (65% in a resistant group and 35% in a susceptible group) [51], and Central Java [50]. In Asia, a high frequency of the mutant allele V1016G is also common, including in Myanmar (84.4% [46]), and in southern China (95.5% [66]). The V1016G mutation was detected at a lower frequency in Thailand [47], Vietnam [46] and Malaysia [48].

For locus 1534, the homozygous wild-type genotype was frequent at all collection sites. A high frequency of the wild-type genotype was also found in *Ae. aegypti* mosquitoes from Jakarta (70.70%), Central Java (91.16%) [50] and Denpasar (63.91%) [52]. The 1534C allele frequency in this study (8%) was lower compared to the neighbouring countries of Southeast Asia, including Malaysia (40-80%) [48], Vietnam (22%) [46], Myanmar (21%) [46] and Thailand (51%) [67].

In the present study, there were three patterns of co-occurrence of point mutations, namely, V1016G/F1534C, V1016G/S989P and V1016G/F1534C/S989P. We found the simultaneous occurrence of *kdr* mutations V1016G and F1534C in all collection sites. Homozygous mutants at locus 1016 had homozygous wild-type alleles at locus 1534 and *vice versa*, and heterozygous V1016G were also heterozygous for F1534C, apart from a small number of combined homozygous/heterozygous genotypes. The main co-occurrence was homozygous mutants at 1016 and homozygous wild-type alleles at 1534 (1016G/F1534 haplotype) at a frequency of 82.1%. Pearson’s correlation analysis strongly supports statistical associations between mutations in all collection sites. Correspondingly, a similar linkage association is also found in the *Ae. aegypti* population from our previous study, where these genotypes occurred at 69.2% [49], and from Myanmar where the equivalent frequency was 59.3% [46]. In contrast, the more common association in *Ae. aegypti* from Thailand was a homozygous F1534C mutation with V1016G wild-type at frequency 43.5% [47]. Double mutants of 1016G and 1534C were never found. This is consistent with other studies, which have noted that in natural populations of *Ae. aegypti*, the double homozygotes for V1016G and F1534C mutations are rare [46–49] or absent [47], suggesting a possible fitness cost related to this haplotype or absence of recombination to date to bring homozygotes for V1016G and F1534C mutations onto the same haplotype [54]. However, a small number of mosquitoes homozygous for 1016G and 1534C occur in Malaysia [48] and Myanmar [46]. Diverse haplotypes associated with V1016G and F1534C co-occurrences across the countries suggest that selection has acted differently across sites perhaps related to pyrethroid usage, or else that different genetic backgrounds have had an impact on evolutionary trajectories [68]. Given the linkage disequilibrium between V1016G and F1534C, there is a non-random association of alleles at the two *kdr* loci which may indicate a selection process acting simultaneously at these two loci.

The co-occurrence of the S989P mutation with the V1016G mutation has been noted in previous studies from some other Asian countries [46, 66, 68–70]. However, it also appears that the S989P mutation can be found in the absence of the V1016G mutation. A small number of homozygous and heterozygous mutants at S989P were found to coexist with the F1534C mutation instead of the V1016G mutation in *Ae. aegypti* samples from Yogyakarta outer city areas [49] and Central Java [50]. Additionally, the S989P mutation has been reported as occurring alone in samples from Central Java [50]. The most common tri-locus genotype co-occurrences were homozygous mutant 1016GG and homozygous wild-type FF1534, combined with homozygous mutant 989PP (GG/FF/PP), followed by heterozygous S989P (GG/FF/SP). These results are somewhat different from our previous findings, which detected the most common combination being the homozygous mutant 1016G and homozygous wild-type F1534, combined with heterozygous S989P which was recorded at 39.75% in the resistant group, 26.83% in the susceptible group, and 31% in the field collection [49]. These differences may reflect local selection processes for resistance that differ between the city and outer areas of Yogyakarta.

**Fig. 5:**
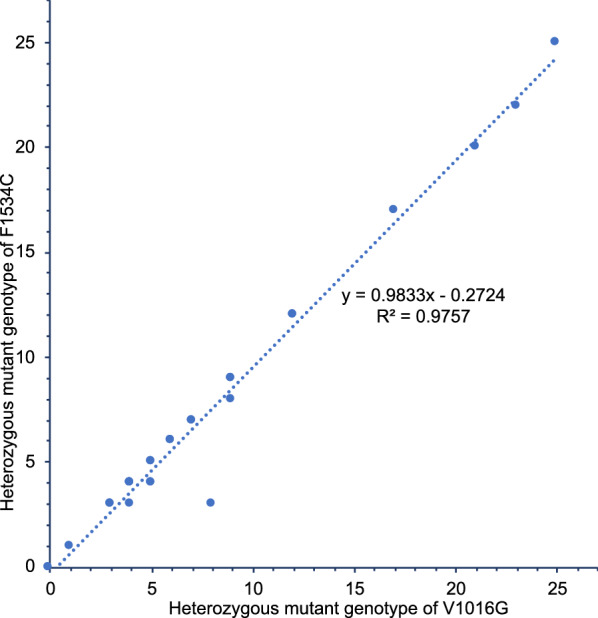
Correlation between the number of *Aedes aegypti* individuals with heterozygous mutant genotype V1016G and heterozygous mutant genotype F1534C across sampling areas

The spatial variation in resistance allele frequencies found in this study may have minor implications for *Wolbachia* mosquito trials being undertaken in the Yogyakarta area [56]. At present, some areas of the city have been invaded by *Wolbachia* whereas other areas are acting as uninvaded controls. The expectation is that dengue incidence will be lower in invaded areas because there is no local transmission or at least reduced transmission where *Wolbachia* is common, based on the fact that the *w*Mel strain released in *Ae. aegypti* mosquitoes partially blocks dengue transmission [71]. However, if there are differences in pesticide resistance across areas, then this may confound interpretations because increased resistance may be associated with a higher local density of mosquitoes which could influence transmission as well. This problem will be alleviated to some extent by backcrossing the *Wolbachia* mosquito release strain to the local wild mosquitoes, so that the local insecticide resistance genetic profile is introgressed to the release strain [56]. Given the relatively small differences in frequency of resistance alleles, we do not anticipate large confounding effects of locality variation in resistance, but it is nevertheless worthwhile controlling for such an effect when comparing impacts of *Wolbachia* (Fig. 5).

Finally, the data suggest that there is no spatial structure to variation in resistance mutations across the city area. This seems to point to local selection events driving resistance frequencies rather than migration at this scale. Still, any attempts to manage pesticide resistance such as through rotating active ingredients will need to be mindful of selection pressures that can locally increase resistance and aim to alter behaviour across the entire inner-city area.

## Conclusions

This study identified *kdr* mutations in *Ae. aegypti* from some areas in the inner city of Yogyakarta managed for *Wolbachia* releases. We detected the highest frequency of the V1016G mutation, and the lowest frequency of the F1534C mutation. The low frequency of the F1565C mutation in the samples may indicate that Type I pyrethroids have not been used as extensively as Type II pyrethroids. We also observed the pattern of the simultaneous occurrence of *kdr* mutations V1016G and F1534C: the V1016/C1534 double homozygote (VV/CC); the G1016/C1534 double heterozygote (VG/FC) and the G1016/F1534 homozygote (GG/FF); and a small amount of combined heterozygous/ homozygous (VG/FF) genotypes. Given the linkage disequilibrium between V1016G and F1534C, there is a non-random association of alleles at the two *kdr* loci which may indicate a selection process acting simultaneously at these two loci. The most common tri-locus genotype co-occurrences were a homozygous mutant 1016GG and a homozygous wild-type FF1534, combined with a homozygous mutant 989PP (GG/FF/PP). This is a bit different from the co-occurrence from Yogyakarta outer city samples which was a homozygous mutant 1016G and a homozygous wild-type F1534, combined with a heterozygous S989P. These differences are perhaps due to their having different local selection processes for resistance between the city and outer areas of Yogyakarta. The relatively low spatial variation in resistance allele frequency across the city may have minor implication for *Wolbachia* mosquito trials.

## Data Availability

Data are presented in the main manuscript.
